# Butyrate Mitigates Lipopolysaccharide-Induced Intestinal Morphological Changes in Weanling Piglets by Regulating the Microbiota and Energy Metabolism, and Alleviating Inflammation and Apoptosis

**DOI:** 10.3390/microorganisms10102001

**Published:** 2022-10-10

**Authors:** Yunsheng Han, Chaohua Tang, Qingyu Zhao, Shijie Fan, Peilong Yang, Junmin Zhang

**Affiliations:** 1 Key Laboratory of Feed Biotechnology of Ministry of Agriculture and Rural Affairs, Institute of Feed Research, Chinese Academy of Agricultural Science, Beijing 100081, China; 2 State Key Laboratory of Animal Nutrition, Institute of Animal Sciences of Chinese Academy of Agricultural Sciences, Beijing 100193, China; 3 Scientific Observing and Experiment Station of Animal Genetic Resources and Nutrition in North China of Ministry of Agriculture and Rural Affairs, Institute of Animal Science of Chinese Academy of Agricultural Sciences, Beijing 100193, China

**Keywords:** coated butyrate, piglet, intestinal morphology, jejunal microbiota, apoptosis, inflammation, energy metabolism

## Abstract

Butyrate provides energy for colonocytes and is a functional metabolite that mitigates weanling piglet stress. However, its effects and mechanisms remain largely unknown. We established a lipopolysaccharide (LPS)-induced inflammatory stress piglet model to examine how butyrate mechanisms impacted piglet intestinal histology, microbiota, and inflammation. We randomly assigned 18 crossbred male piglets to three treatment groups: CON, LPS, and BT-LPS. Coated butyrate was supplemented in the BT-LPS feed for 21 days. On days 19 and 21, piglets in LPS and BT-LPS groups were challenged with LPS at 100 μg/kg body weight. Dietary butyrate improved LPS-injured intestinal histology by significantly increasing jejunal and ileal villus height, villus height to crypt depth ratios, and decreasing histological scores. LPS challenge activated hypoxia-inducible factor 1α and nuclear factor-κB, and enhanced interleukins (IL-1β, IL-6, IL-12), tumor necrosis factor-α, and also downstream inducible nitric oxide synthase and cyclooxygenase 2, but decreased anti-inflammatory cytokines (IL-10, IL-13). Most molecule levels were significantly reversed by butyrate administration. When compared with the CON or LPS groups, the BT-LPS group had a higher relative abundance of jejunal *Firmicutes*, *Bacteroidetes*, *Clostridiaceae*, *Lactobacillus*, and *Prevotella* but a lower abundance of *Proteobacteria*, *Enterobacteriaceae,* and *Escherichia–Shigella*. Phylogenetic investigation of communities by reconstruction of unobserved states and correlation analyses suggested these bacteria contributed to butyrate-alleviating jejunal inflammation and infectious diseases. Butyrate-based diets significantly reduced apoptosis via mitochondrial pathways by downregulating apoptotic *caspase 3* mRNA levels. Diets also altered enterocyte metabolism in the jejunum by upregulating peroxisome-proliferator-activated receptor α expression but downregulating carnitine palmitoyltransferase 1 level when compared with CON or LPS groups. Butyrate supplementation improved immunity homeostasis, generated beneficial shifts in microbial communities, improved enterocyte energy metabolism, and prevented apoptosis to protect intestinal histology from LPS-induced injury.

## 1. Introduction

Modern pig production faces many challenges, especially during weanling piglet transition periods. Weanling stress commonly arises due to shifts from liquid to solid feeding, environment changes, and mixing with new mates [1]. This stress generally induces a post-weaning growth lag, diarrhea, or even a high incidence of mortality and morbidity, leading to economic losses [2]. A crucial factor during stress processes is gastrointestinal tract underdevelopment, with poor digestive and nutrient absorption abilities. Additionally, weanling piglets are vulnerable to intestinal infections with Gram-negative enterotoxigenic *Escherichia coli* pathogens, as well as resultant intestinal immune system activation [3]. This activation induces diverse inflammation cell infiltration and proinflammatory cytokine production involving interleukin 1β (IL-1β), IL-6, and tumor necrosis factor-α (TNF-α), which cause mucosal dysfunction and injury [4]. Lipopolysaccharides (LPS) are composed of lipids and polysaccharides, are derived from Gram-negative bacteria outer membranes, and are strong inflammatory stimulants [5]. LPS interacts with toll-like receptor 4 (TLR4) and triggers inflammatory signaling cascades by activating nuclear factor-κB (NF-κB) and inducing its nuclear translocation, ultimately leading to proinflammatory cytokine secretion and inflammatory responses in piglets [6]. LPS challenge doses, duration, and associated mechanisms are well characterized in animals, and therefore LPS-induced intestinal inflammation models are commonly used to study in vivo inflammatory injury [7,8].

Recently, a previous study has reported close relationships between intestinal microbiota, intestinal mucosa health, and piglet health [9]. In piglet intestines, several lymphoid tissue types are present, including highly organized lymphoid follicles, mesenteric lymph nodes, isolated lymphoid follicles, and lymphocytes, which are influenced by microbiota in terms of intestinal immune function. Healthy microbial communities not only resist pathogen infection but also coordinate balanced anti- and pro-inflammatory responses [6,10]. Intestinal microbiota also regulate intestinal immune systems via flagellin, LPS, and secreted metabolites to accelerate immune system development and maturity [11]. Conversely, intestinal microbial disorders are related to intestinal inflammation [12]; however, excessive inflammatory responses exacerbate intestinal dysfunction and injury, and negatively impact piglet growth and health [13]. This evidence highlights the central role of intestinal microbiota in regulating immune functions and improving piglet health.

Butyrate is primarily derived from the microbial fermentation of dietary fiber and provides approximately 70% of energy requirements for colonocytes [14,15]. In general, butyrate exerts positive effects on livestock production, as well as multiple beneficial effects toward animal physiology. Previously, diets containing sodium butyrate significantly decreased the incidence of post-weaning diarrhea in piglets, enhanced humoral immunity levels by increasing immunoglobulin concentrations, and improved intestinal mucosa integrity [16]. Another study reported that tributyrin-supplemented diets alleviated intestinal injury and enhanced tight-junction formation in piglets [17]. The evidence also suggested that butyrate elevated enterocyte proliferation but decreased apoptosis [18]. Moreover, butyrate regulated intestinal immunity homeostasis by directly acting on immune mucosa cells, increasing T_reg_ numbers and activity, and decreasing neutrophils, macrophages, and effector T cells [19]. A previously characterized butyrate mechanism showed that associated anti-inflammatory properties were promoted via G-protein-coupled receptor 109A signaling [11]. However, the application of sodium butyrate in livestock is generally restricted in practice because of its offensive odor, leading to an adverse effect on feed intake [1,20]. In addition, sodium butyrate is absorbed immediately at the anterior part of the gastrointestinal tract, which limits the efficiency of sodium butyrate absorption in the entire digestive tract [21,22]. Coated sodium butyrate is composed of a secondary coating of sodium butyrate with different purity via intelligent microencapsulation technology that overcomes the aforementioned limits of ordinary sodium butyrate [23]. This microencapsulated coated sodium would slowly and progressively release its active ingredients in the whole intestinal tract and display its function [21,24]. We hypothesized that coated butyrate would improve intestinal injury by shifting intestinal microbiota, regulate signal molecules related to inflammation and apoptosis in LPS-challenged piglets, and ameliorate host intestinal health.

To address these questions, we used an LPS-induced inflammatory stress piglet model to investigate underlying coated butyrate protective mechanisms toward intestinal histology when challenged by LPS. Several key physiological traits were examined: intestinal morphology, jejunal microbiota, inflammation, and enterocyte apoptosis and metabolism. Our data provided a theoretical dietary basis for butyrate in weaned piglets to improve overall host health.

## 2. Materials and Methods

### 2.1. Animals and Experimental Design

We randomly assigned 18 crossbred male weanling piglets (Duroc × Landrace × Yorkshire) with a similar initial body weight (BW = 9.10 ± 0.15 kg) to three treatments. Each treatment comprised six replicates (pens), with one piglet/pen. Treatments included a basal diet and a butyrate diet (basal diet + 3.00 g/kg coated butyrate). The test period was 21 days. The day of weaning was the first day of the experiment, and on the 19th and 21st days of the experiment, the piglets in the CON group were treated by intraperitoneal injection of sterile saline, and piglets in LPS and BT-LPS groups were treated by LPS and then labeled CON, LPS, and BT-LPS. The LPS (from *E. coli* serotype 055:B5; Sigma-Aldrich, St. Louis, MO, USA) was challenged at 100 μg/kg BW in sterile saline one time [7]. A non-medicated basal diet was formulated to address all weanling piglet nutrient requirements according to the National Research Council recommendations [25] (Table 1). Coated butyrate was supplemented as a partially coated sodium butyrate formulation composed of 70% sodium butyrate and 30% fat that was encapsulated using intelligent microencapsulation technology. The dose was based on previous studies [26,27].

Piglets were housed alone in one pen (1.8 × 0.8 m), and adjacent pens were separated by a closed baffle. A hard plastic and fully slotted floor was used in the pen. The room was environmentally controlled; the temperature was controlled at 28–30 °C and 26–28 °C between days 1–14 and 15–21, respectively. During the experimental period, pigs had ad libitum access to food and water.

### 2.2. Sample Collection

On day 21, 12-h-fasted piglets were humanely slaughtered at 4 h after LPS challenge by means of an anesthetic injection. The intestines were removed and divided into the duodenum, jejunum, and ileum sections. Middle duodenum, jejunum, and ileum in 1 cm long intestinal canal sections were obtained; washed lightly in saline; fixed in 4% formalin; and stored at 4 °C. Fresh jejunal mucosa and chyme were collected as previously described [28], immediately frozen in liquid nitrogen, and stored at −80 °C for biochemical analysis.

### 2.3. Intestinal Morphology

Duodenum, jejunum, and ileum specimens were prepared and embedded in paraffin, sectioned at 4 μm, and subjected to hematoxylin and eosin (H&E) and periodic acid–Schiff staining. Three intact crypt villi in one sample were selected, and villus height (VH) and its adjoining crypt depth (CD) were measured with an image analysis system (Version 1, Leica Imaging Systems Ltd., Cambridge, UK) [2]. VH was determined from the apex to the base of the villus crypt junction, and CD was the invagination depth between adjacent villi [29]. VH to CD (V/C) ratios were also calculated. Histological observations were conducted according to the previous study, and histological scores were blindly analyzed using a 0–30 score system for each parameter [30].

### 2.4. Terminal Deoxynucleotidyl Transferase Nick end Labeling (TUNEL)

Jejunum sections were prepared and stained using a commercial TUNEL kit (Fluorescein, Basel, Switzerland, cat. no. 11684817910) [31]. The TUNEL-positive nuclei fluoresced green under a fluorescence microscope, while all nuclei in the jejunum fluoresced blue. Images were taken at high magnification (200×), where five sections were selected from each group for statistical analysis. Apoptotic index = number of TUNEL-positive nuclei/total number of nuclei × 100% [31].

### 2.5. Jejunal Microbiota and Analysis

Bacterial DNA was extracted from jejunal contents with a Qiagen DNA stool mini kit (Qiagen, Hilden, Germany). DNA quantity and quality were determined using a NanoDrop 2000 spectrophotometer (Thermo Fisher Scientific, Waltham, MA, USA) and 1% agarose gels, respectively. The specific hypervariable region (V3–V4) *16S rRNA* was amplified using the primers (forward 5′-ACTCCTACGGGAGGCAGCA-3′ and reverse 5′-GGACTACHVGGGTWTCTAAT-3′) with unique barcodes. Polymerase chain reaction (PCR) was applied in a total volume of 20 µL (including 0.2 μM each primer, 250 μM dNTP, 1 × FastPfu buffer, 1 U FastPfu polymerase (Beijing TransGen Biotech, Beijing, China), and 10 ng template DNA). PCR products were electrophoresed on 2% agarose gels and purified using a commercial kit (Qiagen, Hilden, Germany). Sequencing libraries were constructed using a TruSeq^®^ DNA PCR-Free Sample Preparation Kit (Illumina, San Diego, CA, USA) on the basis of the manufacturer’s recommendations. Library quality was determined using a Qubit V.2.0 Fluorometer (Thermo Fisher Scientific, Waltham, MA, USA). Qualified DNA libraries were loaded into a NovaSeq platform with a capability of 2 × 250 bp paired-end sequencing reads (Novogene, Beijing, China).

Paired-end reads were generated and merged using FLASH software (V1.2.7, http://ccb.jhu.edu/software/FLASH/, accessed on 20 May 2022). Operational taxonomic units with 97% identity were gathered using Uparse (ver. 7.1, http://drive5.com/uparse/, accessed on 20 May 2022). Taxonomic annotations were performed using the Mothur algorithm (70% confidence) in the Silva database (http://www.arb-silva.de/, accessed on 20 May 2022). Alpha-diversity was analyzed using Chao 1 (http://www.mothur.org/wiki/Chao, accessed on 20 May 2022) and Shannon (http://www.mothur.org/wiki/Shannon, accessed on 20 May 2022) indices. Beta-diversity was visualized using principal coordinate analysis (PCoA) plots based on weighted and unweighted Unifrac distance. Bacterial biomarkers between groups were displayed using the linear discriminant analysis effect size (LEfSe) with linear discriminant analysis (LDA) > 3.5. Phylogenetic investigation of communities by reconstruction of unobserved states (PICRUSt) was used to assess metagenome function on the basis of different Kyoto Encyclopedia of Genes and Genomes (KEGG) levels [32]. The relative percentage of predictive pathways at KEGG level II was presented as a heatmap with normalized analysis.

### 2.6. Biochemical Analysis

Jejunum mucosa (appropriately 100 mg) samples were mixed and homogenized in 1 mL precooled saline using an Ultra-Turrax homogenizer (Scientz, Ningbo, China) and then vortexed for 30 s. Homogenates were centrifuged at 3000 × *g* for 15 min at 4 °C, and supernatants were transferred to new tubes for protein assay and other measurements. Immune indices were measured using enzyme-linked immunosorbent assay kits (Shanghai Enzyme-linked Biotechnology Co., Ltd., Shanghai, China) according to the manufacturer’s protocols. Assay molecules included hypoxia-inducible factor 1α (HIF-1α), nuclear factor-κB (NF-κB), NF-κB p65, interleukins (IL-1β, IL-6, IL-8, IL-10, IL-12, IL-13, IL-17), tumor necrosis factor α (TNF-α), transcription growth factor β (TGF-β), cyclooxygenase 2 (COX-2), and inducible nitric oxide synthase (iNOS). Inflammatory related parameters were normalized to tissue protein concentrations, which were measured using a bicinchoninic acid commercial kit (Thermo Fisher Scientific, Waltham, MA, USA).

### 2.7. Real-Time Quantitative PCR (RT-qPCR)

Selected mRNA abundance was determined by RT-qPCR, including apoptosis genes *Bcl-2*, *Bak*, *Bax*, *caspase 3*, *caspase 8*, and *caspase 9* and energy metabolism genes acyl-CoA oxidase 1 (*ACOX1*), succinate dehydrogenase (*SDH*), peroxisome-proliferator-activated receptor α (*PPARα*), nuclear respiratory factor 1 (*NRF1*), uncoupling protein 2 (*UCP2*), mitochondrial transcription factor A (*TFAM*), α-ketoglutarate-dependent dioxygenase (*FTO*), malic enzyme 1 (*ME1*), and carnitine palmitoyltransferase 1 (*CPT1*). Total RNA was isolated from jejunum samples (approximately 0.75 mg) using an RNAprep pure tissue kit (Tiangen Biotech Co. Ltd., Beijing, China). Total RNA concentrations and quality were estimated using a NanoDrop 2000 spectrophotometer (Thermo Fisher Scientific, Waltham, MA, USA), which confirmed that high-quality absorbance ratios (260/280 nm) were between 1.8 and 2.0. RNA integrity was evaluated using agarose gel (1%) electrophoresis. Then, cDNA was synthesized from 1 μg total RNA using a PrimeScript RT reagent kit (TaKaRa Biotechnology Co., Ltd., Otsu, Japan) according to the manufacturer’s instructions. Selected mRNA reactions were detected in 10 µL (ABI Prism 7700 Sequence Detection System; Applied Biosystems, Foster City, CA, USA) using SYBR^®^ Premix Ex Taq^TM^ II (Tli RNaseH Plus) (TaKaRa Biotechnology Co., Otsu, Japan). The primers for energy metabolism, apoptosis-related, and housekeeping genes (glyceraldehyde 3-phosphate dehydrogenase (GAPDH)) were described previously [31,33]. The 2^−ΔΔCt^ method was used for quantification using *GAPDH* as a reference gene, and relative abundance was normalized to CON group values.

### 2.8. Statistical Analysis

Statistical analyses were performed using one-way analysis of variance in SAS 9.4 (SAS Institute, Inc., Cary, NC, USA). Each piglet served as a statistical unit. Differences between groups were evaluated using Duncan’s multiple-range tests. Tukey’s HSD tests were used to test for differences in microbial diversity. Metastat and LEfSe analyses were used to test for significant differences between microbiota relative abundance. Results were represented as the mean with standard error of mean (SEM) in the tables and the mean with standard error (SE) in the figures, while *p* < 0.05 and *p* < 0.01 values were considered statistically and extremely significant, respectively. Bar charts were drafted in Graphpad Prism 7.0 software (GraphPad Software Inc., La Jolla, CA, USA).

## 3. Results

### 3.1. The Effects of Butyrate on LPS-Challenged Intestinal Morphology in Piglets

Piglets in BT-LPS and LPS groups had lower VH and V/C duodenum values when compared to the CON group (*p* < 0.05, Table 2). Compared with the CON group, the LPS challenge significantly reduced VH jejunum and ileum values and V/C ileum ratios (*p* < 0.05), while butyrate administration significantly increased these parameters (*p* < 0.05). Jejunum CD values in BT-LPS and LPS groups were lower than the CON group (*p* < 0.05), and V/C ratios in the BT-LPS group were higher than the LPS group (*p* < 0.05).

### 3.2. The Effects of Butyrate on LPS-Challenged Jejunum Histological Scores

**Figure 1:**
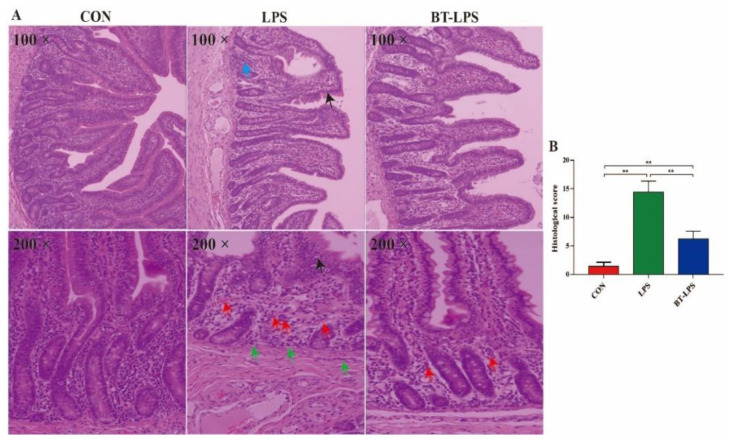
The effects of butyrate on LPS-induced jejunal histology in weaned piglets. (**A**) Representative jejunum histological sections of LPS-induced and butyrate-treated piglets. (**B**) Jejunal histological scores on day 21 based on a 0–30 score evaluation system. Arrows in images indicate major histological differences between treatments: intraepithelial lymphocytes (black arrows), granulocyte inflammation (blue arrow), neutrophil inflammation (red arrows), monocyte inflammation (green arrows). Values are the mean ± standard error (*n* = 5). ** *p* < 0.01.

Histological jejunum examinations indicated that LPS challenge increased mucosal intraepithelial lymphocytes (black arrows), as well as granulocyte infiltration and crypt damage (blue arrows) (Figure 1). Extensive inflammatory cell infiltration comprising neutrophils in the mucosa (red arrows) and monocytes in the submucosa (green arrows) were identified in LPS-challenged piglets. Dietary butyrate improved these parameters as indicated by histological scores. Piglets in the BT-LPS group had lower histological scores when compared with LPS animals but higher histological scores when compared to the CON group (*p* < 0.05).

### 3.3. The Effects of Butyrate on LPS-Challenged Jejunum Inflammation

**Figure 2:**
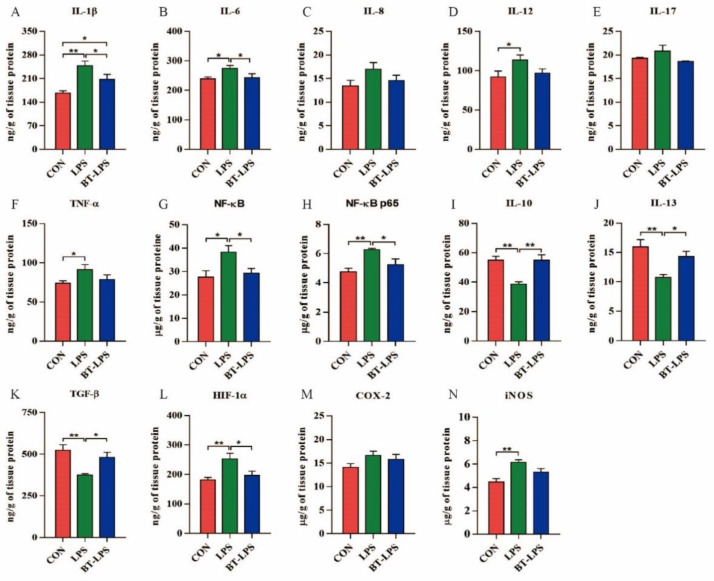
The effects of butyrate on LPS-induced jejunal inflammation. (**A**–**H**) The pro-inflammatory associated cytokine IL-1β, IL-6, IL-8, IL-12, IL-17, and TNF-α levels; (**I**–**K**) The anti-inflammatory cytokine IL-10, IL-13, and TGF-β levels; (**G**,**H**) The transcription factor NF-κB and NF-κB p65 levels; and (**L**–**N**) HIF-1α, COX-2, and iNOS levels in LPS-induced jejunum. Values are the mean ± standard error (*n* = 6). * *p* < 0.05 and ** *p* < 0.01.

LPS challenge induced inflammation in jejunum tissue (Figure 2). When compared with the CON group, LPS animals displayed significantly increased pro-inflammatory-associated elements/cytokines IL-1β, IL-6, IL-12, TNF-α, NF-κB, and NF-κB p65 (*p* < 0.05, Figure 2A–H), while butyrate diets significantly decreased IL-1β, IL-6, NF-κB, and NF-κB p65 levels when compared with the LPS animals (*p* < 0.05). IL-1β level in the BT-LPS group was significantly higher than the CON group (*p* < 0.05). Anti-inflammatory cytokine IL-10, IL-13, and TGF-β levels in LPS animals were significantly higher than in CON and BT-LPS animals (*p* < 0.05, Figure 2I–K), but no differences were observed between BT-LPS and CON groups (*p* > 0.05). Piglets in the LPS group had higher HIF-1α and iNOS levels when compared with the CON group (*p* < 0.05, Figure 2L,N), while BT-LPS animals had lower HIF-1α levels when compared with the LPS group (*p* < 0.05).

### 3.4. The Effects of Butyrate on Microbiota Diversity in Jejunum Exposed to LPS

**Figure 3:**
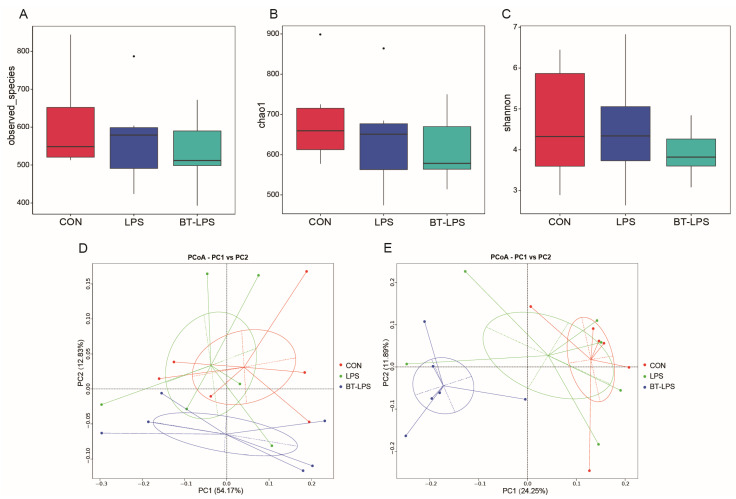
The effects of butyrate on microbiota diversity in LPS-induced jejunum. (**A**–**C**) Bacterial richness and diversity were estimated using Observed_species, Chao 1 value, and Shannon indices; “.” represents outlier points. (**D**,**E**) Principal coordinate analysis based on weighted and unweighted Unifrac distance showed separations among groups. Values are the mean ± standard error (*n* = 6).

In total, 1,074,885 effective sequences (1,520,294 raw reads) were generated from piglet jejunum microbiota samples, with an average of 59,716 sequences/sample. Alpha-diversity analyses showed that a butyrate diet and LPS challenge did not affect bacterial community α-diversity Observed_species, Chao 1, and Shannon indices (*p* > 0.05, Figure 3A–C). Beta-diversity PCoA analyses based on weighted and unweighted Unifrac distance showed that microbial communities were well separated between groups, especially between BT-LPS and the other groups (Figure 3D,E).

### 3.5. The Effects of Butyrate on Bacterial Community Structures, Challenged by LPS, in the Jejunum

**Figure 4:**
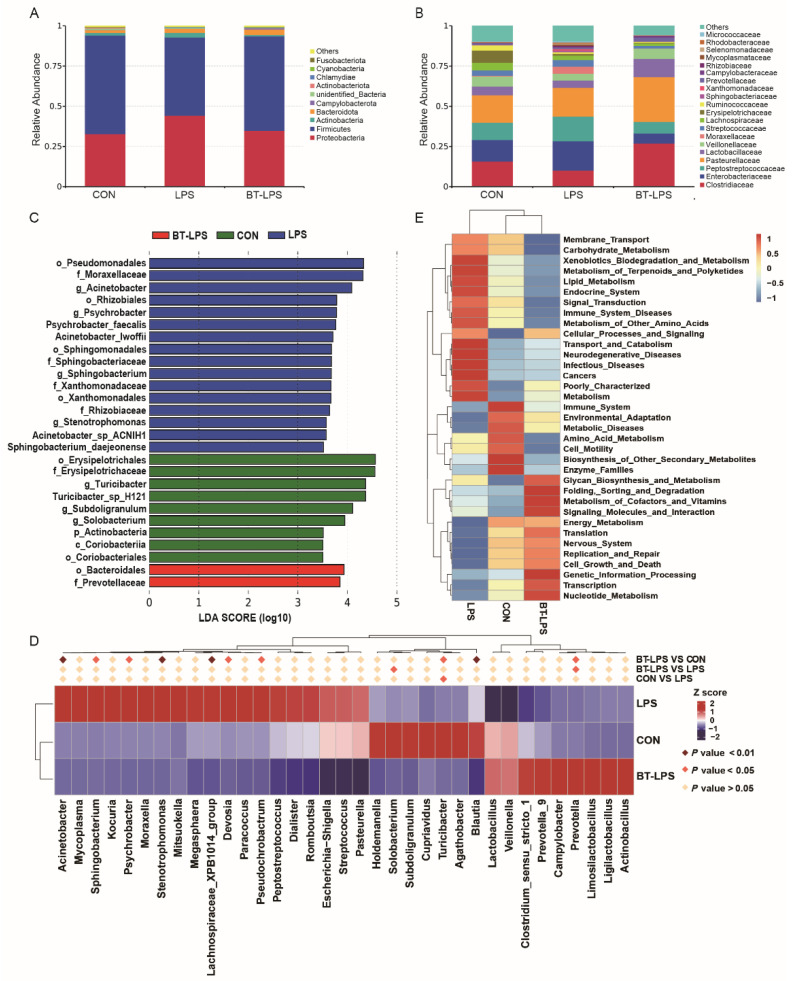
The effects of butyrate on bacterial compositions in LPS-induced jejunum. (**A**,**B**) Distribution of jejunal bacteria at phylum and family levels. (**C**) Taxa enrichment based on LEfSe analysis identified significant microbial community differences between groups. A logarithmic LDA score > 3.5 was selected. (**D**) Metastat was used to test for significant differences between the top 35 genera relative abundance; dark pink diamonds indicate *p* < 0.01, and deep pink diamonds indicate *p* < 0.05 differences between two groups. (**E**) The top 35 predicted metabolism pathways based on KEGG II levels from PICRUSt analysis. Values are the mean ± standard error (*n* = 6).

At the phylum level, *Firmicutes*, *Proteobacteria*, *Bacteroidetes*, and *Actinobacteria* were the four major bacterial phyla in the CON group, with relative abundances of 61.38%, 32.78%, 1.64%, and 1.58%, respectively (Figure 4A). Compared with the CON group, LPS challenge increased the relative abundance of *Proteobacteria*, *Bacteroidetes*, and *Actinobacteria* by 35.18%, 53.05%, and 81.68%, respectively, but decreased the relative abundance of *Firmicutes* by 20.79% (*p* > 0.05). The relative abundances of *Firmicutes*, *Proteobacteria*, *Bacteroidetes*, and *Actinobacteria* were, respectively, 58.81%, 34.77%, 3.38%, and 0.86% in the BT-LPS group, which indicated that butyrate supplementation reduced the relative abundance of *Firmicutes* and *Actinobacteria* by 4.19% and 45.56%, respectively, but increased the relative abundance of *Proteobacteria* and *Bacteroidetes* by 6.07% and 106.10%, respectively, when compared to the CON group (*p* > 0.05).

At the family level, the *Prevotellaceae* relative abundance in the BT-LPS group was significantly higher than the LPS group (*p* < 0.05, Figure 4B), and no significant difference was observed between the CON group and other two groups (*p* > 0.05). The *Clostridiales* relative abundance in the BT-LPS group (26.95%) was higher than LPS (10.11%) and CON (15.71%) groups (*p* > 0.05). The *Lactobacillaceae* relative abundance in the BT-LPS group (11.30%) was higher than the LPS group (4.45%) and even higher than the CON group (5.47%) (*p* > 0.05). When compared to the CON group, LPS challenge increased the relative abundance of *Enterobacteriaceae* from 13.43% to 18.22%, whereas the butyrate diet decreased *Enterobacteriaceae* relative abundance from 18.22% to 6.19% when compared to the LPS group (*p* > 0.05).

LEfSe analyses indicated that *g_Acinetobacter*, *g_Psychrobacter*, *g_Sphingobacterium*, and *g_stenotrophomonas* constituted the dominant bacteria in the LPS animals (Figure 4C). In contrast, in the BT-LPS group, *f_Prevotellaceae* was the dominant bacteria. Of the 35 most dominant genera, the LPS challenge significantly decreased the relative abundance of *Turicibacter* when compared with the CON group (*p* < 0.05, Figure 4D), while butyrate supplementation significantly increased the relative abundance of *Prevotella* when compared with LPS and CON groups (*p* < 0.05). Compared with the CON group, the butyrate diet significantly decreased *Blautia*, *Acinetobacter*, *Psychrobacter*, *Pseudochrobactrum*, *Devosia*, *Stenotrophomonas*, and *Sphingobacterium* relative abundance (*p* < 0.05). The *Escherichia–Shigella* relative abundance in the BT-LPS animals (6.14%) was lower than the CON animals (13.18%) and the LPS animals (16.80%) (*p* > 0.05). PICRUSt analyses suggested that jejunal bacteria in butyrate-fed piglets provided a lower risk of immune system and infectious diseases when compared with LPS or CON groups (Figure 4E).

### 3.6. Correlation Analyses between the Jejunal Microbiota and Immune Indices

**Figure 5:**
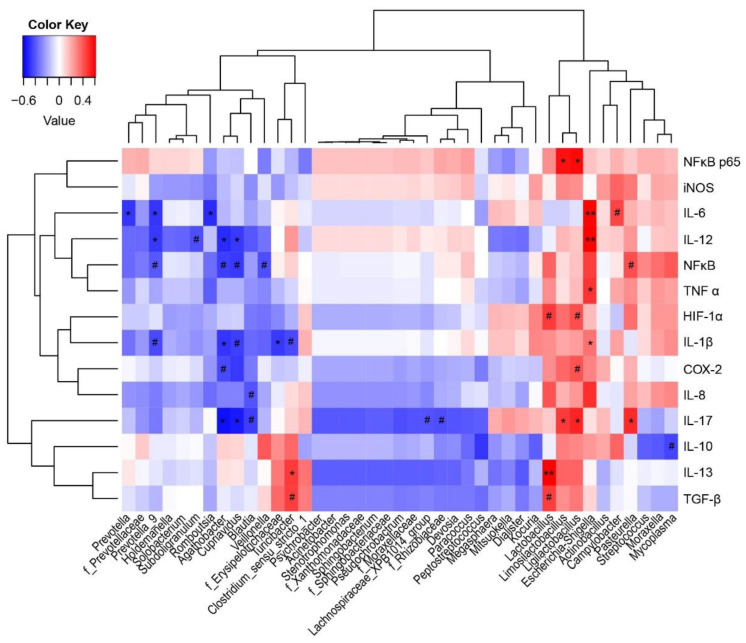
Correlations between jejunal microbiota and immune parameters. Red indicates a positive correlation and blue indicates a negative correlation. * *p* < 0.05 and ** *p* < 0.01 indicate significant and extremely significant correlations, while # 0.05 < *p* < 0.01 represents a correlation trend.

A Spearman’s correlation analysis was used to explore relationships between predominant genera and families and immune-associated parameters (Figure 5). *Lactobacillus* and *Turicibacter* were significantly positively correlated with IL-13 (r = 0.610, 0.468), while *Escherichia–Shigella* was significantly positively correlated with IL-1β (r = 0.332), IL-6 (r = 0.589), IL-12 (r = 0.598), and TNF-α (r = 0.485) levels (*p* < 0.05). *Limosilactobacillus* and *Ligilactobacillus* showed significantly positive correlations with IL-17 (r = 0.468, 0.515) and NF-κB p65 (r = 0.567, 0.578) (*p* < 0.05). *Prevotella* and *Prevotella_9* were significantly negatively correlated with IL-6 (r = −0.480, −0.483), while *Prevotella_9* was significantly negatively correlated with IL-12 (r = −0.471) (*p* < 0.05).

### 3.7. The Effects of Butyrate on Jejunum-LPS Challenged Cell Apoptosis

**Figure 6:**
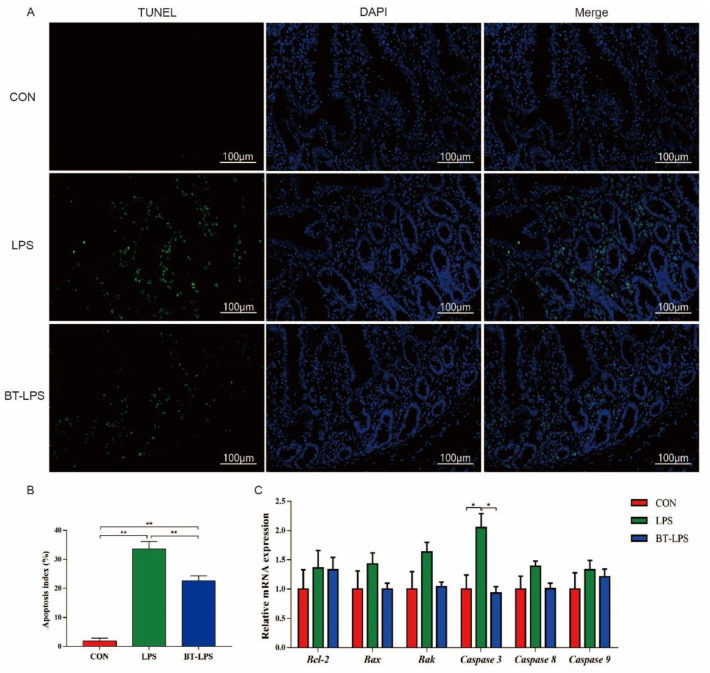
The effects of butyrate on LPS-induced jejunal apoptosis. (**A**,**B**) TUNEL staining of jejunal tissue and the apoptotic index. Values are the mean ± standard error (*n* = 5). (**C**) Relative apoptosis gene mRNA levels in the jejunum (*n* = 6). * *p* < 0.05 and ** *p* < 0.01.

TUNEL staining showed that positive nuclei in the LPS jejunum were significantly higher than in CON and BT-LPS jejuna (*p* < 0.05, Figure 6A,B). Butyrate supplementation significantly decreased these numbers when compared with the LPS group, but they were still significantly higher than in the CON group (*p* < 0.05). *Caspase 3* relative mRNA levels in the LPS group were significantly increased (*p* < 0.05, Figure 6C), while *Bak* relative mRNA levels showed an increased trend (*p* = 0.08) when compared with CON and BT-LPS groups. The transcript abundances of *Bax*, *caspase 8*, and *caspase 9* were 43.0%, 39.0%, and 33.0% greater in the LPS animals, respectively, than in the CON animals (*p* > 0.05), but in the BT-LPS animals, these parameters were 30.1%, 27.3%, and 9.0% lower than LPS animals’ levels, respectively (*p* > 0.05).

### 3.8. The Effects of Butyrate on Energy Metabolism Gene mRNA Levels in LPS-Induced Jejunum

**Figure 7:**
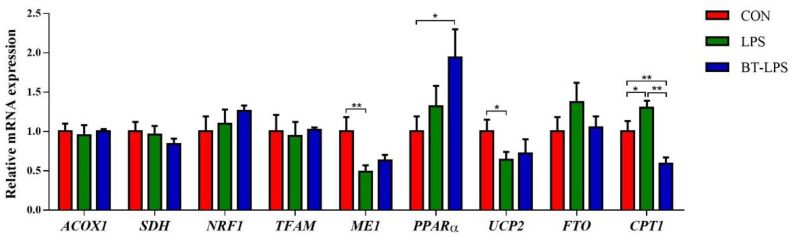
The effects of butyrate on energy metabolism relative gene mRNA levels in LPS-induced jejunum. Values are the mean ± standard error (*n* = 6). * *p* < 0.05 and ** *p* < 0.01.

When compared with the CON group, butyrate supplementation significantly upregulated jejunal *PPARα* mRNA levels (*p* < 0.05, Figure 7), while the LPS challenge significantly decreased *ME1* and *UCP2* mRNA expression (*p* < 0.05). The LPS challenge significantly upregulated *CPT1* mRNA levels when compared to the CON group (*p* < 0.05), but dietary butyrate significantly decreased *CPT1* mRNA expression levels when compared to CON and LPS groups (*p* < 0.05). We observed no significance differences in *ACOX1*, *SDH*, *NRF1*, *TFAM*, and *FTO* transcript abundance between groups (*p* > 0.05).

## 4. Discussion

Growth retardation due to weanling stress is a common problem in weaned piglets. Previously, weanling-associated intestinal inflammation negatively affected epithelial functions in piglets [34]. LPS is a major enterotoxin produced by *E. coli* and damages intestinal functions or even causes piglet diarrhea, which negatively impact growth performance and generate huge economic production losses [8]. For this reason, LPS is widely used to construct acute inflammatory injury piglet models [35]. In our previous study, LPS challenge significantly decreased piglet average daily gain between days 15 and 21 and increased clinical scores between days 19 and 21 when compared with the CON group, while butyrate supplementation significantly decreased these clinical scores when compared with the LPS group [6]. Our observations were similar to a previous study showing that sodium butyrate supplementation (0.2%) to a deoxynivalenol diet improved weaned piglet performance [36]. These data were possibly attributed to improvements in gut health. To further highlight the beneficial effects of butyrate, we studied its effects on intestinal morphology, jejunal microbiota, inflammatory responses, apoptosis, and metabolism in an LPS-induced piglet model.

Intestinal morphology is an important mechanical defense barrier reflecting intestinal health and absorptive capacity. Intestinal morphological alterations, including villus atrophy and crypt hyperplasia, represent malabsorption and body growth retardation [37,38], and decreased VH indicates LPS toxicosis in the intestine [38]. In our study, LPS challenge induced acute intestinal injury, as LPS piglets had lower intestinal VH and V/C ratios when compared with the CON group. These adverse effects on intestinal morphology by LPS were significantly prevented by coated butyrate supplementation, especially for the jejunum and ileum. This was consistent with a previous study that reported that sodium butyrate administration not only increased VH and V/C values but also enhanced intestinal barrier function, as demonstrated by increased transepithelial electrical resistance and decreased fluorescein isothiocyanate dextran 4 kDa flux [39,40]. Thus, butyrate supplementation exerted positive effects on intestinal morphology and contributed to digestive capacity and barrier function. Most probably, butyrate improved homeostatic immunity, as intestinal integrity and epithelial function were negatively affected by inflammatory status [40]. Additionally, butyrate possibly alleviated intestinal mucosa damage induced by noxious bacteria via bacteriostasis, which improved intestinal morphological structures and functions [41].

The jejunum is important in preventing pathogens and toxins from the external environment into the circulation system, and its digestive enzymes and cytokines are associated with growth and immune function [42,43]. Weaned piglets with growth retardation had a higher apoptosis rate in jejunum epithelial cells and pro-inflammatory response than those with normal body weight [44]. The efficiency of glyceryl butyrate to improve enterotoxigenic *Escherichia coli*-induced inflammatory response by NFκB/MAPK pathway in the ileum was not as good as that in the jejunum [45]. In addition, most attention was focused on colonic microbiota, while jejunal microbiota has been shown to be essential for the host’s physiology, which includes metabolic, immune, and endocrine functions [46,47]. The jejunum responds strongly to the involved microbiota, even though there are low bacterial densities and diversity [48]. In a time-course study of metabolic responses in gastrointestinal tract mucosa to conventionalization, a strong and dynamic response was found in the jejunum but not the ileum and colon, indicating the unique role of the jejunum in response to the luminal microbiota [46,49]. Combined with the present result, dietary coated butyrate significantly improved jejunal morphology induced by LPS. Therefore, the jejunum was selected to further measure inflammation, microbiota, apoptosis, and metabolism.

During post-weaning periods, LPS derived from Gram-negative bacteria induced inflammatory responses by activating the TLR 4/NF-κB-mediated pathway, in which the inflammatory response was partly correlated with histology injury [12]. Inflammatory cell activation and mediator release are caused by intestinal barrier dysfunction [50]. In our study, LPS challenge induced severe, higher jejunal histological scores in intraepithelial lymphocyte, granulocyte, neutrophil, and monocyte infiltration. Moreover, LPS stimulation significantly increased pro-inflammatory-associated molecule and cytokine production, such as NF-κB, NF-κB p65, TNF-α, IL-1β, IL-6, IL-12, and IL-17 in the jejunum. These increases suggested that LPS successfully induced inflammatory responses, with the NF-κB pathway having key roles initiating this inflammation. HIF-1α is a transcription factor and is associated with metabolic and inflammatory disorders [31]. HIF-1α also activates the central regulator NF-κB and causes inflammation via inhibitor of NF-κB phosphorylation and the extracellular-signal-regulated kinase1/2-mediated phosphorylation of serine 276 on p65 [51]. However, short chain fatty acids (SCFAs) may decrease pro-inflammatory cytokine secretion by suppressing NF-κB [52]. This observation agreed with our data; coated butyrate significantly diminished HIF-1α increases and associated downstream pro-inflammatory molecules and cytokines, and therefore decreased histological scores induced by LPS. A similar result was observed in a *Clostridium difficile*-challenge mouse study, wherein butyrate significantly reduced histological scores in the colon and alleviated inflammatory responses, as reflected by decreased IL-1β, IL-6, and chemokine ligand 1 secretion [30]. Moreover, a glycerol butyrate diet was shown to modulate jejunal and ileal inflammation, as indicated by the decrease in IL-1β, IL-6, and TNF-α levels [45]. However, in our study, this diet significantly reversed decreases in anti-inflammatory cytokines induced by LPS, including IL-10, TGF-β, and IL-13. T-regulatory cell IL-10 production was recently shown to prevent exaggerated T-cell responses and protect against intestinal inflammation via a self-limiting mechanism [53]. TGF-β positively affected epithelial recovery and improved inflammation, while TGF-β deficiency accelerated immune system dysfunction [54,55]. These observations indicated that butyrate supplementation contributed to homeostatic immunity, where regulatory mechanisms were related to butyric acid and G-protein-coupled receptor interactions or histone deacetylase inhibition [15].

Intestinal microbial communities generally participate in nutrient metabolism, and associated disorders are largely associated with disrupted intestinal digestion, inflammation, and morphology [41,56]. To investigate the effects of coated butyrate on jejunal microbiota in piglets challenged by LPS, we compared microbial diversity and composition between treatment groups. However, no significant differences were observed in α-diversity between groups. As reported, the oral administration of sodium butyrate to weaned piglets decreased microbial diversity in the ileum but increased levels in the colon [57]. This discrepancy may have been due to the type and dose of butyrate and different intestinal segments analyzed. Our β-diversity results showed that microbiota in the BT-LPS group was distinct from microbiota in CON or LPS groups, which suggested coated butyrate altered jejunal microbial structures. Specifically, coated butyrate supplementation increased the relative abundance of *Firmicutes* and *Bacteroidetes* but decreased the relative abundance of *Proteobacteria* and *Actinobacteria* when compared with the LPS group. An increase in *Firmicutes* proportion is beneficial to intestinal health, as it contains abundant SCFA-producing bacteria [8]. *Clostridiaceae*, *Clostridium_sensu_stricto_1*, *Lactobacillaceae*, and *Lactobacillus* were involved in the *Firmicutes,* and their relative abundance was higher in the BT-LPS group when compared with the LPS group and even the CON group. This concurred with a previous study showing sodium butyrate infusion tended to accelerate *Firmicutes* and *Clostridiaceae* proliferation in the colon [58]. Dietary sodium butyrate also increased colonic *Clostridiaceae* relative abundance in weaned piglets [57]. *Lactobacillus* is a commonly used probiotic organism and a characterized bacteriocin and organic acid producer. Compelling previous evidence showed that *Lactobacillus* had excellent probiotic properties in a dextran-sulfate-sodium-induced mice model, where it maintained the intestinal epithelial barrier and alleviated inflammatory responses [59]. Other studies indicated that *Lactobacillus* protected intestinal health via its metabolites, bacteriostasis, and the competitive exclusion of pathogens at mucosa sites [8,60]. As demonstrated in a *Staphylococcus aureus* infection model, *Lactobacillus* supplementation effectively decreased *S. aureus* levels and accelerated intestinal damage recovery [61]. Another study demonstrated that the *Lactobacillus* stress protein GroEL enhanced IL-13 secretion and decreased apoptosis induced by LPS [62]. Additionally, in our study, *Lactobacillus* was significantly correlated with IL-13 levels. This compelling evidence supported the concept that butyrate administration improved intestinal histology and inflammation induced by LPS challenge.

Intestinal dysbiosis is generally associated with increased facultative anaerobic bacteria levels. Facultative anaerobes, *Proteobacteria*, including *Enterobacteriaceae* and *Escherichia–Shigella*, proliferate and interfere with host nutrition by metabolizing fermentation products in oxygenated conditions [63]. *Escherichia–Shigella* is a pathogen that induces intestinal inflammation by activating NF-κB and mitogen-activated protein kinase pathways [12,64]. Shiga toxin produced by *E. coli* promotes fluid and electrolyte accumulation in the bowel by disrupting balances in intestinal absorption and secretion, leading to higher diarrhea incidences and lower piglet growth [57,65]. These observations agreed with our study where an LPS challenge increased the relative abundance of *Escherichia–Shigella*, *Enterobacteriaceae,* and *Proteobacteria*, and caused severe inflammatory responses. However, coated butyrate supplementation decreased this bacterial abundance and decreased pro-inflammatory cytokine levels. This was partly consistent with a previous report showing that sodium butyrate administration inhibited the proliferation of facultative anaerobes [66]. We also showed that *Escherichia–Shigella* significantly positively correlated with TNF α, IL-1β, IL-6, and IL-12 levels, indicating that a butyrate-based diet alleviated intestinal inflammation and was possibly related to a decrease in the relative abundance of *Escherichia–Shigella*. As reported, host protection against intestinal infection mechanisms partly depends on the fight between facultative anaerobic *Enterobacteriaceae* and obligate anaerobic *Clostridiaceae* [67]. This observation was also reflected by our data. A lower acid intestinal environment, due to butyrate supplementation, probably suppressed the colonization of these pathogens [68]. Another reason could be iNOS levels, which were higher in the LPS animals when compared to the CON animals but were non-statistically reduced in the BT-LPS animals. Decreased iNOS levels were shown to control facultative anaerobic bacteria by mitigating nitric oxide conversion to nitrate, thus reducing levels of additional electron acceptors [69].

*Bacteroidota*, including *Prevotellaceae*, *Prevotella*, and *Prevotella_9*, are well known for modulating piglet growth due to energy derived from bacterial fermentation [57]. *Prevotella* promotes complex dietary polysaccharide conversion to monosaccharides for host uptake and confers performance advantages [70,71]. Additionally, *Prevotella* metabolizes plant cell walls and produces high SCFA levels, which are later absorbed by enterocytes to promote intestinal immunity homeostasis [72]. In our study, coated butyrate supplementation significantly increased the relative abundance of *Prevotella* when compared with the CON and LPS groups, while *Prevotella* or *Prevotella_9* were significantly negatively correlated with IL-6 and IL-12 levels. This was partly explained by the fact that butyrate supplementation alleviated intestinal inflammation, and possibly explained why piglets in the BT-LPS group had better growth performances and clinical scores in our previous study [6]. These findings suggested that dietary coated butyrate improved intestinal injury and appeared to be related to a microbiota shift. Combined PICRUSt and correlation analyses indicated that coated butyrate supplementation alleviated intestinal inflammation, partly by accelerating the prevalence of *Firmicutes*, *Bacteroidetes*, *Clostridiaceae*, *Lactobacillus*, and *Prevotella* but partly suppressing *Proteobacteria*, *Enterobacteriaceae*, and *Escherichia–Shigella* proliferation.

Apoptosis is a form of physiological cell death that controls epithelial turnover in the intestinal mucosa. Pathogens and associated toxins induce cell damage and apoptosis, causing villus atrophy during piglet weaning periods [4]. This observation was consistent with our TUNEL staining data, which indicated that an LPS challenge significantly increased jejunal cell apoptosis, with upregulated *caspase 3* and *Bax* expression. Apoptosis is an important mechanism suppressing enterocyte proliferation. Generally, mitochondrial apoptosis pathways are modulated by the Bcl-2 family, the caspase family, apoptosis-induce factor, and second mitochondria-derived activator of caspases [31,73]. A previous study reported that a deoxynivalenol challenge caused intestinal epithelial cell apoptosis by upregulating Bax and caspase 3 protein expression [9]. However, our coated-butyrate-based diet significantly mitigated LPS-induced apoptosis/as reflected by TUNEL-positive cell levels and *caspase 3* and *Bak* expression. These observations concurred with a previous study showing that diet with 0.1% tributyrin improved apoptosis and intestinal injury [17]. The anti-apoptotic butyrate effects may be closely correlated with the butyrate-mediated alleviation of intestinal inflammation induced by LPS challenge. As reported, inflammation usually promotes small intestinal cell apoptosis [74,75]. Another probable mechanism could be that butyrate protects against apoptosis by inhibiting endoplasmic reticulum stress, which is regulated by protein-kinase-like endoplasmic reticulum kinase-eukaryotic initiation factor 2-α pathway via GPR109A [76]. Collectively, these results indicated that coated butyrate supplementation effectively ameliorated LPS-induced intestinal apoptosis and injury and was reflected by improved intestinal histology.

Our data also indicated that coated butyrate protection against LPS-induced apoptosis was mediated by a mitochondria-dependent pathway. Energy metabolism in intestinal enterocytes was measured by gene expression. ME1 is an important enzyme required for lipid synthesis as well as being a malate metabolizing enzyme that catalyzes the reversible oxidative decarboxylation of L-malate coupled to the reduction of dinucleotide cofactor nicotinamide adenine dinucleotide phosphate to yield pyruvate and carbon dioxide [33,77]. *PPARα* is a master regulator involved in *β*-oxidation and controls mitochondrial *β*-oxidation by modulating its downstream target gene carnitine palmitoyltransferase 1 (*CPT1*) [78]. In our study, coated butyrate supplementation and LPS challenge significantly increased *PPARα* and *CPT1* mRNA levels, respectively, when compared with the CON group. This suggested that fatty acids were mobilized to provide energy for intestinal enterocyte proliferation during LPS challenge. Moreover, LPS challenge significantly decreased *ME1* expression, with no differences observed between BT-LPS and CON groups. This suggested that fatty acid metabolism in the BT-LPS group was more balanced between synthesis and catabolism. As reported, CPT1 is a rate-limiting enzyme of fatty acid oxidation and controls long-chain fatty acid entry into the mitochondria [79]. This potentially explained our data, showing that coated butyrate administration significantly decreased CPT1 expression when compared with the LPS group, as CPT1 is not required for shuttling SCFA esters into mitochondrial oxidation. *UCP2* has important roles in fatty acid metabolism and reduces reactive oxygen species during mitochondrial electron transport [80]. Significantly lower *UCP2* expression is associated with mitochondrial dysfunction [81]. These observations were consistent with our results, showing that coated butyrate supplementation appeared to mediate significant reductions in *UCP2* expression caused by LPS challenge, which may contribute to the butyrate alleviation of jejunal apoptosis induced by LPS.

## 5. Conclusions

**Figure 8:**
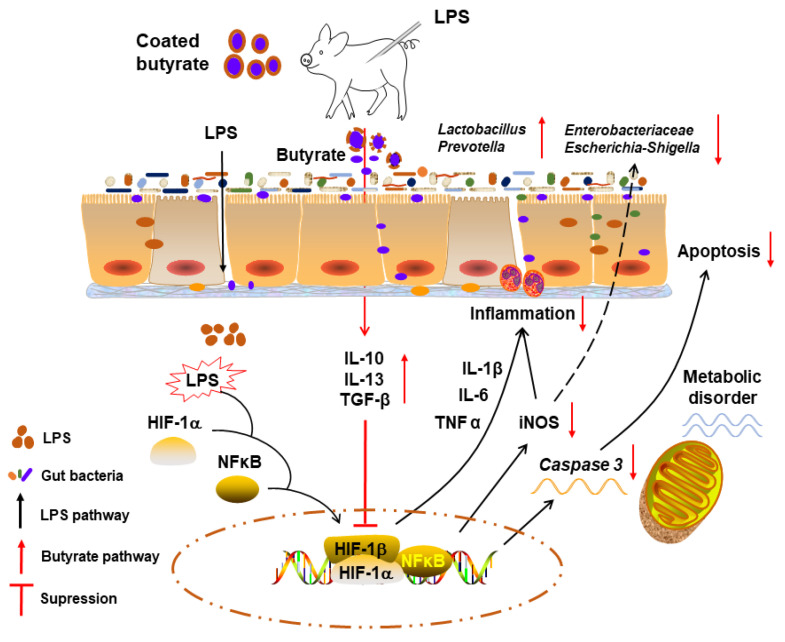
Schematic showing how butyrate putatively protects weanling piglets from LPS-induced intestinal histology changes by regulating microbiota and energy metabolism levels, as well as alleviating inflammation and apoptosis.

We showed that coated butyrate significantly increased intestinal VH and V/C ratios, decreased jejunal histological scores, and improved jejunal homeostatic immunity by enhancing anti-inflammatory and reducing pro-inflammatory cytokines in an LPS-induced piglet model. Dietary butyrate optimized LPS-induced microbial disorders by increasing the prevalence of jejunal *Firmicutes*, *Bacteroidetes*, *Clostridiaceae*, *Lactobacillus*, and *Prevotella,* as well as suppressing *Proteobacteria*, *Enterobacteriaceae,* and *Escherichia–Shigella*. Additionally, butyrate supplementation alleviated jejunal apoptosis and shifted enterocyte energy metabolism at the mRNA level. Therefore, a butyrate-based diet protected piglet intestinal histology from LPS-induced injury by improving homeostatic immunity, microbial community, and enterocyte energy metabolism, and prevented jejunal apoptosis (Figure 8). Our data provided a theoretical basis for protecting weanling stress in piglets using coated butyrate.

## Figures and Tables

**Table 1:** Ingredients and chemical composition of experimental diets (as-fed basis).

Ingredient (%)	Content
Extruded corn	55.25
Soybean meal	11.30
Extruded soybean	10.00
Fish meal	5.00
Soybean protein concentrate	4.00
Whey powder	8.00
Sucrose	2.00
Soy oil	1.50
Dicalcium phosphate	1.00
Limestone	0.50
Sodium chloride	0.20
L-Lysine-HCl	0.30
DL-Methionine	0.20
L-Threonine	0.15
L-Tryptophan	0.10
Premix ^†^	0.50
Nutrient composition ^‡^	
Digestible energy (MJ/kg)	14.50
Crude protein (%)	19.10
Calcium (%)	0.82
Total phosphorus (%)	0.72
Available phosphorus (%)	0.49
SID * lysine (%)	1.23
SID * methionine (%)	0.36
SID * threonine (%)	0.74
SID * tryptophan (%)	0.20

^†^ Provided per kg of diet: vitamin A, 2200 IU; vitamin D_3_, 220 IU; vitamin E, 11 IU; vitamin K_3_, 0.5 mg; vitamin B_12_, 0.015 mg; riboflavin, 4 mg; niacin, 30 mg; pantothenic acid, 10 mg; choline chloride, 400 mg; folic acid, 0.3 mg; thiamine, 1.5 mg; vitamin B_6_, 3 mg; biotin, 0.1 mg; zinc, 100 mg; manganese, 4 mg; iron, 84 mg; copper, 6 mg; iodine, 0.14 mg; and selenium, 0.35 mg. ^‡^ Nutrient levels are calculated. * SID = standardized ileal digestibility. The feed formulation was cited from Han et al. (2020) [6].CC.BY.

**Table 2:** The effects of butyrate on intestinal morphology in piglets challenged with LPS.

Item	Dietary Treatment ^1^			SEM ^2^	*p*-Value
	CON	LPS	BT-LPS		
Duodenum					
Villus height (μm)	391.43 ^a^	274.29 ^b^	284.76 ^b^	8.960	0.002
Crypt depth (μm)	333.21	291.43	306.67	6.633	0.464
Villus height/crypt depth	1.16 ^a^	0.93 ^b^	0.90 ^b^	0.029	<0.001
Jejunum					
Villus height (μm)	348.19 ^a^	281.24 ^b^	334.38 ^a^	6.408	<0.001
Crypt depth (μm)	281.52 ^a^	242.66 ^b^	252.38 ^b^	4.292	<0.001
Villus height/crypt depth	1.25 ^ab^	1.16 ^b^	1.34 ^a^	0.025	0.018
Ileum					
Villus height (μm)	344.29 ^a^	265.90 ^b^	325.05 ^a^	7.472	<0.001
Crypt depth (μm)	269.33	250.47	253.90	3.484	0.059
Villus height/crypt depth	1.28 ^a^	1.06 ^b^	1.28 ^a^	0.024	<0.001

^a,b^ Mean values within a row with different superscript letters indicate significant differences between groups (*p* < 0.05). ^1^ CON, control group, basal diet; LPS, lipopolysaccharide group, basal diet, and intraperitoneally challenged with LPS; BT-LPS, butyrate-LPS group, basal diet supplemented with coated butyrate at 3.0 g/kg, and intraperitoneally challenged with LPS. ^2^ SEM, standard error of the mean, *n* = 6.

## Data Availability

The datasets generated for this study can be found in the NCBI sequence read archive, accession number PRJNA648691.
